# The Effect of Brain–Computer Interface Training on Rehabilitation of Upper Limb Dysfunction After Stroke: A Meta-Analysis of Randomized Controlled Trials

**DOI:** 10.3389/fnins.2021.766879

**Published:** 2022-02-07

**Authors:** Weiwei Yang, Xiaoyun Zhang, Zhenjing Li, Qiongfang Zhang, Chunhua Xue, Yaping Huai

**Affiliations:** Rehabilitation Department, Shenzhen Longhua District Central Hospital, Shenzhen, China

**Keywords:** brain-computer interface, upper extremity, rehabilitation, stroke, meta-analysis

## Abstract

**Background:**

Upper limb motor dysfunction caused by stroke greatly affects the daily life of patients, significantly reduces their quality of life, and places serious burdens on society. As an emerging rehabilitation training method, brain–computer interface (BCI)–based training can provide closed-loop rehabilitation and is currently being applied to the restoration of upper limb function following stroke. However, because of the differences in the type of experimental clinical research, the quality of the literature varies greatly, and debate around the efficacy of BCI for the rehabilitation of upper limb dysfunction after stroke has continued.

**Objective:**

We aimed to provide medical evidence-based support for BCI in the treatment of upper limb dysfunction after stroke by conducting a meta-analysis of relevant clinical studies.

**Methods:**

The search terms used to retrieve related articles included “brain-computer interface,” “stroke,” and “upper extremity.” A total of 13 randomized controlled trials involving 258 participants were retrieved from five databases (PubMed, Cochrane Library, Science Direct, MEDLINE, and Web of Science), and RevMan 5.3 was used for data analysis.

**Results:**

The total effect size for BCI training on upper limb motor function of post-stroke patients was 0.56 (95% CI: 0.29–0.83). Subgroup analysis indicated that the standard mean differences of BCI training on upper limb motor function of subacute stroke patients and chronic stroke patients were 1.10 (95% CI: 0.20–2.01) and 0.51 (95% CI: 0.09–0.92), respectively (*p* = 0.24).

**Conclusion:**

Brain–computer interface training was shown to be effective in promoting upper limb motor function recovery in post-stroke patients, and the effect size was moderate.

## Introduction

Stroke is the leading cause of death and disability worldwide, and the 2016 Global Burden of Disease (GBD) study highlighted that Chinese people have a lifetime risk of stroke of up to 39.3%, which is the highest in the world (GBD 2016, 2018). Furthermore, persistent impairment of upper limb movement is one of the most common disabilities for patients following stroke, which seriously impact patients' daily lives (Broeks et al., 1999; Bhatnagar et al., 2020); two-thirds of patients have upper limb dysfunction 6 months after the onset of stroke (Dobkin, 2005).

To date, various rehabilitation techniques have been proposed for the rehabilitation of post-stroke patients, which include physical therapy, occupational therapy, constraint-induced motor therapy, neuromuscular electrical stimulation, and task-oriented training (Veerbeek et al., 2014; Lin et al., 2019). Most rehabilitation strategies focus mainly on behavioral training and not directly on the brain. Moreover, the effect of conventional rehabilitation on the sequelae of cerebral infarction, such as hypokinesia and poor coordination, is usually unsatisfactory. Therefore, strategies that allow direct stimulation of the brain, such as transcranial magnetic stimulation and transcranial direct current stimulation, show promise for achieving more effective outcomes.

Recent developments in the field of biomedical engineering and rehabilitation robots have led to the introduction of brain–computer interfaces (BCIs) for stroke rehabilitation. Typically, BCI systems allow the completion of specific actions independent of cerebral electricity outputs to peripheral nerves and skeletal muscles (Wolpaw et al., 2000). BCI training is composed of three modules: signal acquisition, signal processing, and interactive control. Through BCI training, patients can directly control external devices with their brain and produce corresponding movements. According to the signal source, common control modes of BCIs can be categorized into steady-state visual evoked potential, motor imagined rhythm signal, P300 potential, and mixed BCI (Yu, 2017). Furthermore, they can be classified into two general categories: auxiliary and rehabilitative BCI, depending on the forms of use. Auxiliary BCI is a commonly used paradigm in clinical work, which involves the application of BCIs to rehabilitation robots, artificial limbs, and other devices to help patients carry out daily living activities to improve their quality of life. In contrast, rehabilitative BCI acquires patients' neural signals in real time through the BCI and provides feedback training according to the signal processing results, which provides the closed-loop rehabilitation training mode from the central to the peripheral. In general, BCI training requires patients to maintain a high level of concentration during training, which enhances neural plasticity owing to the numerous repeated central stimulus feedback (Yulian and Sijie, 2020). Thus, BCI allows patients to engage in safe, standard, and repeatable rehabilitation training with maximum participation.

The clinical practice of neural rehabilitation is based on the hypothesis that motor learning promotes motor recovery after stroke (Kitago and Krakauer, 2013; Maier et al., 2019). BCI activates neural recovery through motor imagination and motion observation (da Silva et al., 2020) and ensures that patients' motor intention is well-matched with the auxiliary means during the training process to complete the “central-peripheral-central” closed-loop pathway and achieve an effective training effect (Mengya et al., 2019). Recently, several studies reported that BCI training is beneficial to the recovery of upper limb function after stroke (Baniqued et al., 2021). However, the results of these experiments vary and have limited significance for clinical applications. Therefore, we analyzed several randomized clinical trials (limited to clinical trials involving non-invasive BCI) to provide evidence-based support for BCI for the rehabilitation of upper limb dysfunction in post-stroke patients.

## Materials and Methods

### Search Strategy

The review included randomized controlled trials (RCTs) that investigated the effect of BCI-based training on the rehabilitation of upper limb function in post-stroke patients. Search terms that included “upper extremity,” “stroke,” and “brain-computer interface” were used to query several databases, including PubMed, Cochrane Library, Science Direct, MEDLINE, and Web of Science to retrieve relevant articles. Only English articles published up until March 26, 2021, were included. We also checked the reference lists of the articles to retrieve additional relevant articles for the analysis.

### Inclusion and Exclusion Criteria

The retrieved articles were independently screened by two researchers (WY and YH) by reviewing the titles and abstracts using the same inclusion and exclusion criteria. After eliminating repetitive articles using the Endnote software, we reviewed the titles and abstracts of each article to exclude review articles, non-English articles, case reports, conference minutes, and books. If we could not clearly understand the type of study by the abstract, we read the article in its entirety to avoid missing relevant research. Discrepancies were resolved by consensus with a third reviewer (CX).

The inclusion criteria for selecting the articles were as follows: (1) all stroke patients were diagnosed with confirmation by CT or MRI; (2) stroke patients had sequelae of upper limb dysfunctions; (3) control group also underwent evaluation of the effects of the BCI group and other routine rehabilitation; (4) none of the patients had cognitive impairment; (5) study design was an RCT.

The exclusion criteria were as follows: (1) studies that included patients with comorbidities of unstable tachyarrhythmia, fever, infection, seizures, or sedative use; (2) reviews, abstracts, case reports, or non-clinical studies; (3) studies that were not written in English; (4) insufficient data reported even after attempting to contact the corresponding author; (5) duplicated articles.

### Data Extraction and Quality Assessment

The extracted information included first author name, publication year, number of participants, participant characteristics (age and sex), intervention received and time of intervention, and outcome indicators [Fugl–Meyer Assessment Scale of Upper Extremity (FMA-UE) and Modified Function Test (MFT)]. Articles that could not be classified according to the title and abstract alone were retrieved as full texts. If there were disagreements, the two authors (XZ and ZL) discussed the article with a third party (QZ) to reach a consensus. Because all the studies were RCTs, the Cochrane Handbook for Systematic Reviews of Interventions was used to assess the quality of the included studies. The criteria comprise seven elements: random sequence generation, allocation concealment, blinding of participants and personnel, blinding of outcome assessment, incomplete outcome data, selective reporting, and other sources of bias. Two researchers (CX and QZ) independently read the full text of the article to assess the quality based on the seven elements. If the study met all of the conditions, it was considered “Grade A”; if it only met some of the conditions, it was classified as “Grade B”; if the study met none of the conditions, it was considered “Grade C.” If there was a conflict in grade, the quality of the article was decided following a discussion.

### Outcome Indicators

Outcome indicators used in our study were FMA-UE and MFT. FMA-UE is now widely used in the clinical assessment of motor function. Previous studies have shown that FMA-UE is reliable, effective, and feasible for the evaluation of post-stroke upper limb function (Platz et al., 2005; Amano et al., 2018; Hijikata et al., 2020). However, several studies (Jang et al., 2016) used MFT as the primary evaluation standard instead of FMA-UE. Therefore, we used both the FMA-UE and MFT to calculate the pooled effect size.

### Statistical Analysis

Data analysis was performed using the Review Manager software version 5.3 (a software from Cochrane Informatics and Knowledge Management Department). The data analyzed were the changes in patients from baseline to after treatment. For data collection, we calculated the mean differences between pre- and post-intervention for each study according to the Cochrane Handbook for Systematic Reviews of Interventions guidelines. The *I*^2^ statistic was used to test the heterogeneity of the studies. If *p* > 0.05 and *I*^2^ ≤ 50%, this indicated no heterogeneity among studies, and a fixed-effect model was selected for further analysis. If *p* ≤ 0.05 and *I*^2^ > 50%, this indicated significant heterogeneity among studies, and a random-effects model was used for statistical analysis. If heterogeneity could not be ignored, we conducted a sensitivity analysis to determine the source of the heterogeneity. In our study, all data were continuous variables; thus, we used the standard mean difference (SMD) method. Moreover, because the included studies used different evaluation criteria, we used random-effects models for the analysis. For the subgroup analysis of intervention time, we used a random-effects model. A *p*-value < 0.05 was considered statistically significant.

## Results

### Search Results

**Figure 1:**
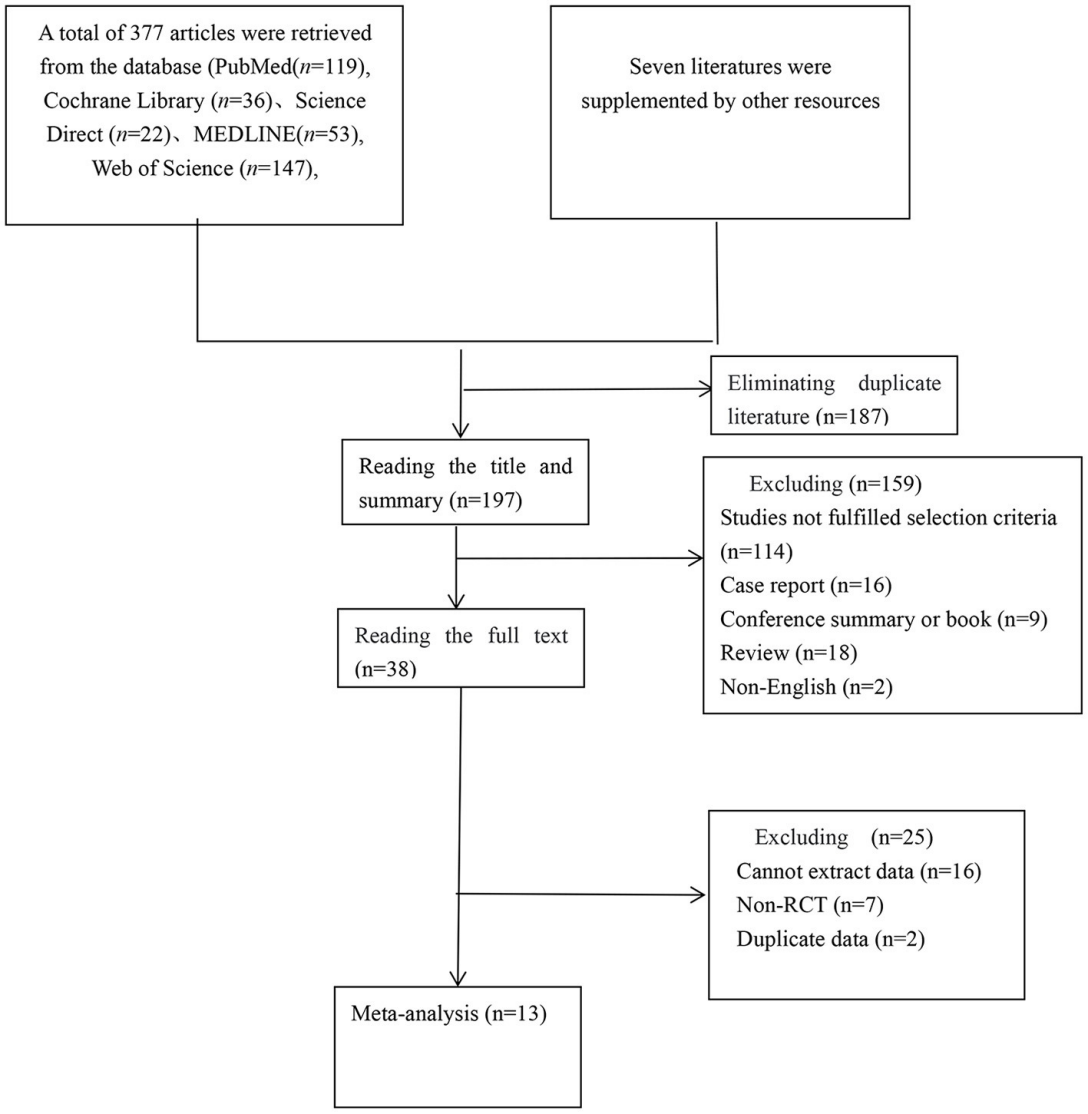
Flow chart of study selection.

A total of 384 articles were reviewed: 119 studies from PubMed, 36 studies from Cochrane Library, 22 studies from ScienceDirect, 53 studies from MEDLINE, 147 studies from Web of Science, and 7 studies from other sources. We eliminated 187 duplicate studies. The independent screening of titles and abstracts resulted in the exclusion of 18 review articles, two non-English studies, 16 case reports, and nine conference summaries or book chapters. Seventy-two studies were excluded because they compared BCI systems rather than investigating the clinical effect or were not BCI training interventions. Another 52 studies did not meet our requirements or included healthy subjects as controls. Some experiments included in the 23 articles were excluded because the data or full text could not be extracted or the study was a cross-control trial. In addition, two studies were excluded because they utilized the same data. Finally, 13 studies that comprised 258 patients were included in our meta-analysis based on the Preferred Reporting Items for Systematic Reviews and Meta-Analyses (PRISMA) protocol. The detailed literature retrieval process is shown in Figure 1 and Table 1 (Ramos-Murguialday et al., 2013; Ang et al., 2014, 2015; Li et al., 2014; Pichiorri et al., 2015; Jang et al., 2016; Biasiucci et al., 2018; Lin et al., 2018; Chen et al., 2020; Cheng et al., 2020; Lee et al., 2020; Miao et al., 2020; Wu et al., 2020).

**Table 1:** Characteristics of the RCTs.

**References**	**Subjects**		**Age (years)**		**Type of interventions**		**Time of interventions**	**Outcome measures**
	**T**	**C**	**T**	**C**	**T**	**C**		
Ang et al. (2014)	6	7	54.0 ± 8.9	58 ± 19.3	EEG-BCI + routine rehabilitation	Routine rehabilitation	1.5 h/d, 3 d/wk, 6 wk	FMA-UE
Ang et al. (2015)	11	14	48.5 ± 13.5	53.6 ± 9.5	BCI	Routine rehabilitation	1.5 h/d, 3 d/wk, 4 wk	FMA-UE
Biasiucci et al. (2018)	14	13	56.4 ± 9.9	59.0 ± 12.4	EEG-BCI + FES	Sham stimulation	1 h/d, 2 d/wk, 5 wk	FMA-UE, MFT, MRC
Jang et al. (2016)	10	10	61.10 ± 13.77	61.70 ± 12.09	BCI+FES	FES	20 min/d, 5 d/wk, 6 wk	MFT, MAS
Lee et al. (2020)	13	13	55.15 ± 11.57	58.30 ± 9.19	EEG-BCI-FES + routine rehabilitation	Routine rehabilitation	30 min/d, 5 d/wk, 4 wk	FMA-UE, MAL, MBI, ROM
Ramos-Murguialday et al. (2013)	16	14	49.3 ± 12.5	50.3 ± 12.2	EEG-BCI	Routine rehabilitation	40 min/d, 5 d/wk, 20 d	FMA-UE, MAS, GAS
Wu et al. (2020)	14	11	62.93 ± 10.56	64.82 ± 7.22	BCI	Routine rehabilitation	1 h/d, 5 d/wk, 4 wk	FMA-UE, ARAT, WMFT
Miao et al. (2020)	8	8	48.80 ± 16.70	50.3 ± 17.1	BCI + routine rehabilitation	Routine rehabilitation	3 sessions/wk, 4 wk	FMA-UE
Lin et al. (2018)	5	5	45.0 ± 11.2	49.0 ± 10.8	BCI + MTD-VR	MTD-VR	35 min/d, 3 d/wk, 4 wk	FMA-UE
Chen et al. (2020)	7	7	41.6 ± 12.0	52.0 ± 11.1	MI-BCI	MI	3 sessions/wk, 4 wk	FMA-UE
Li et al. (2014)	7	7	66.29 ± 4.89	66.00 ± 6.30	BCI + routine rehabilitation	Routine rehabilitation	1.5 h/d, 3 d/w, 8 wk	FMA-UE, ARAT
Cheng et al. (2020)	5	5	62.4 ± 4.7	61.4 ± 4.5	BCI + soft robotic	Soft robotic	90 min/session, 3 sessions/wk, 6 wk	FMA-UE, ARAT
Pichiorri et al. (2015)	14	14	64.1 ± 8.4	59.6 ± 12.7	MI-BCI	MI	30 min/d, 3 d/wk, 4 wk	FMA-UE, MAS, MRC

*T, experimental group; C, control group; FMA-UE, Fugl–Meyer Assessment Scale of Upper Extremity; MFT, Modified Function Test; MRC, Medical Research Council Scale; MAS, Modified Ashworth Scale; MBI, Modified Barthel Index; ROM, range of motion; GAS, Goal Attainment Scale; ARAT, Action Research Arm Test; WMFT, Wolf Motor Function Test*.

### Quality Assessment

**Figure 2:**
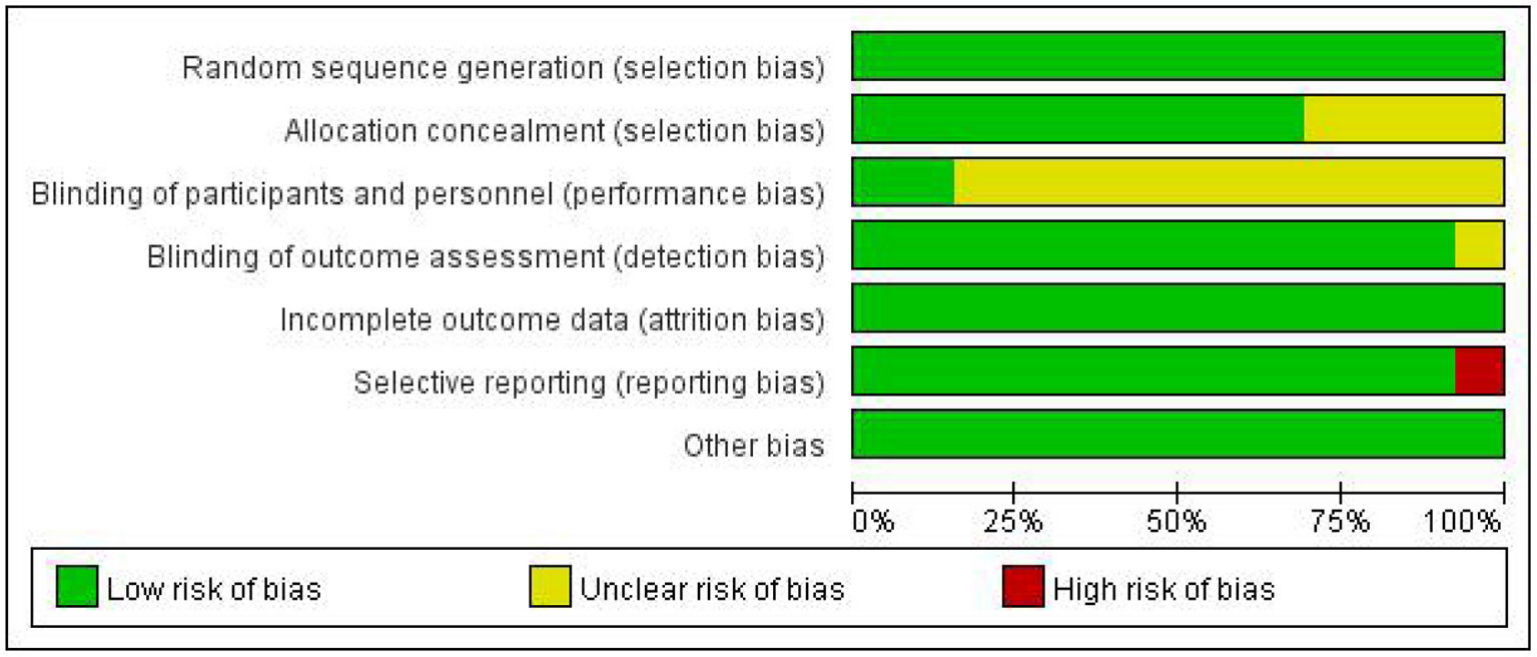
Risk of bias graph.

**Figure 3:**
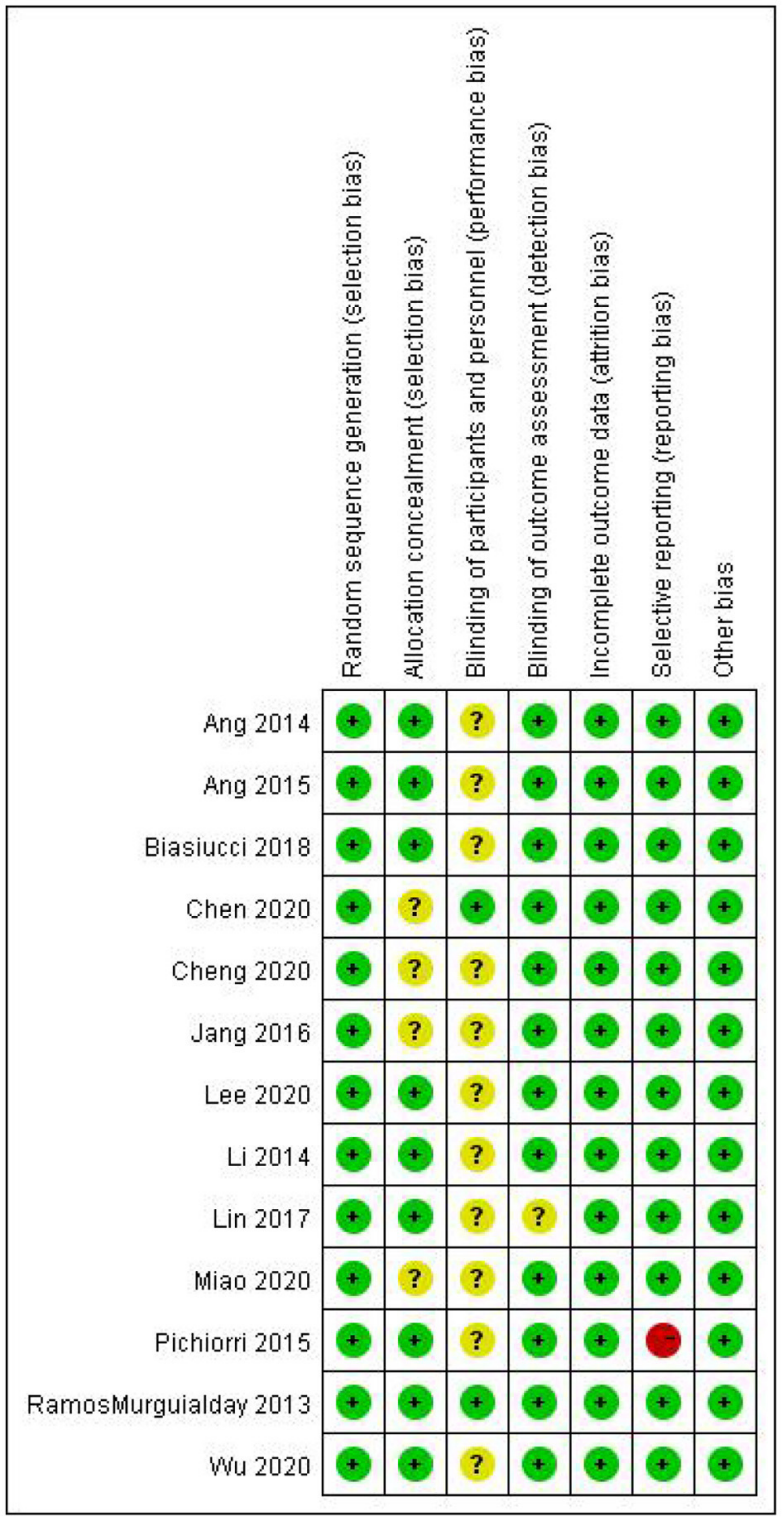
Risk of bias summary.

The quality assessment of the included RCTs is shown in Table 2 and Figures 2, 3. A total of 130 BCI subjects and 128 patients receiving traditional treatments from 13 studies were included in the final analysis. Among the 13 studies, one was considered to have an evidence level of “Grade A,” and the other 12 studies were considered to have an evidence level of “Grade B.” The studies that were categorized as “Grade B” involved selective reporting, an unrigorous design, and a non-blind method. The study with the highest evidence level was that by Ramos-Murguialday et al. (2013), and the study with the lowest was by Pichiorri et al. (2015). Consistency analysis was conducted on the basic information of patients across all studies, and the differences were not significant.

**Table 2:** Methodological quality assessment of the RCTs.

**References**	**Random sequence generation**	**Allocation concealment**	**Blinding of participants and personnel**	**Blinding of outcome assessment**	**Incomplete outcome data**	**Selective reporting**	**Other bias**	**Grade**
Ang et al. (2014)	Low risk	Low risk	Unclear	Low risk	Low risk	Low risk	Low risk	B
Ang et al. (2015)	Low risk	Low risk	Unclear	Low risk	Low risk	Low risk	Low risk	B
Biasiucci et al. (2018)	Low risk	Low risk	Unclear	Low risk	Low risk	Low risk	Low risk	B
Jang et al. (2016)	Low risk	Unclear	Unclear	Low risk	Low risk	Low risk	Low risk	B
Lee et al. (2020)	Low risk	Low risk	Unclear	Low risk	Low risk	Low risk	Unclear	B
Ramos-Murguialday et al. (2013)	Low risk	Low risk	Low risk	Low risk	Low risk	Low risk	Low risk	A
Wu et al. (2020)	Low risk	Low risk	Unclear	Low risk	Low risk	Low risk	Low risk	B
Miao et al. (2020)	Low risk	Unclear	Unclear	Low risk	Low risk	Low risk	Low risk	B
Lin et al. (2018)	Low risk	Low risk	Unclear	Unclear	Low risk	Low risk	Low risk	B
Chen et al. (2020)	Low risk	Unclear	Low risk	Low risk	Low risk	Low risk	Low risk	B
Li et al. (2014)	Low risk	Low risk	Unclear	Low risk	Low risk	Low risk	Low risk	B
Cheng et al. (2020)	Low risk	Unclear	Low risk	Low risk	Low risk	Low risk	Low risk	B
Pichiorri et al. (2015)	Low risk	Low risk	Unclear	Low risk	Low risk	High risk	Low risk	B

### Efficacy of BCI

#### Efficacy of BCI on Upper Limb Motor Function

**Figure 4:**
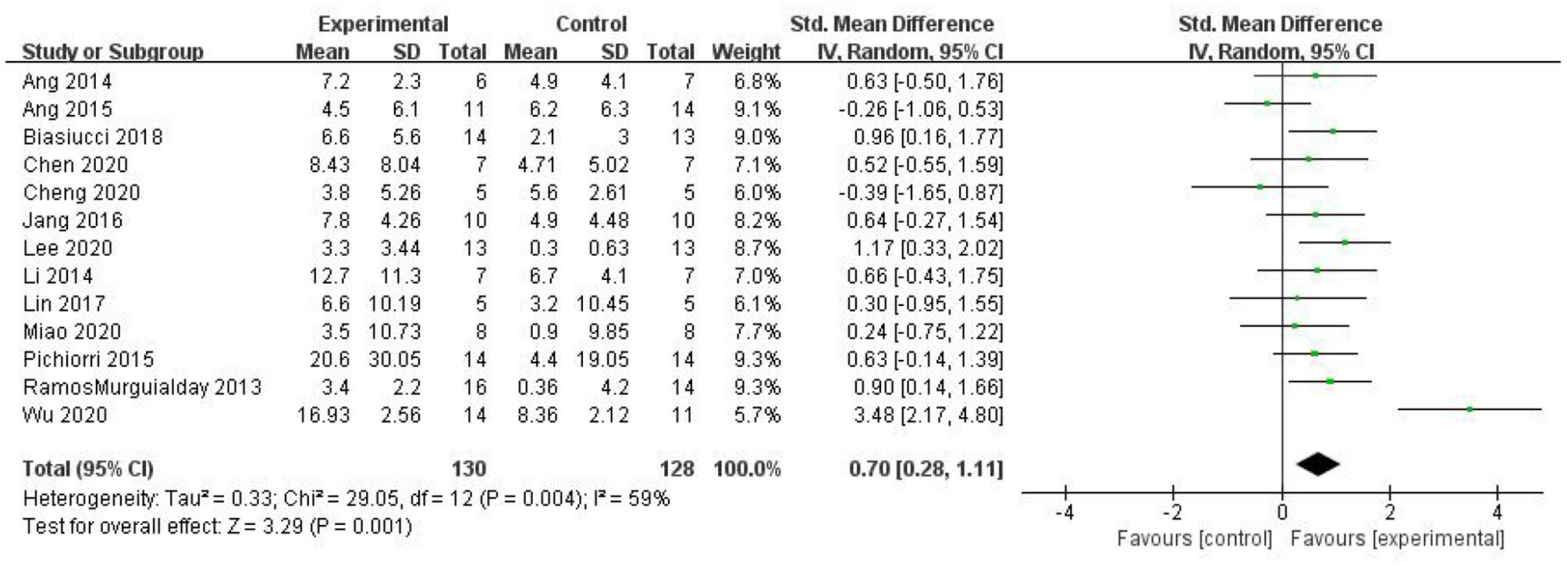
Comparison of the effects of BCI interventions and control interventions on upper limb dysfunction before sensitivity analysis.

**Figure 5:**
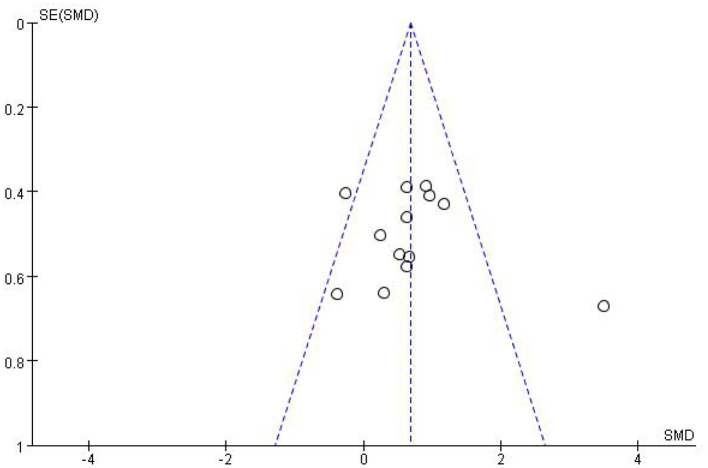
Funnel plot of comparison of the effects of BCI interventions and control interventions on upper limb dysfunction before sensitivity analysis.

**Figure 6:**
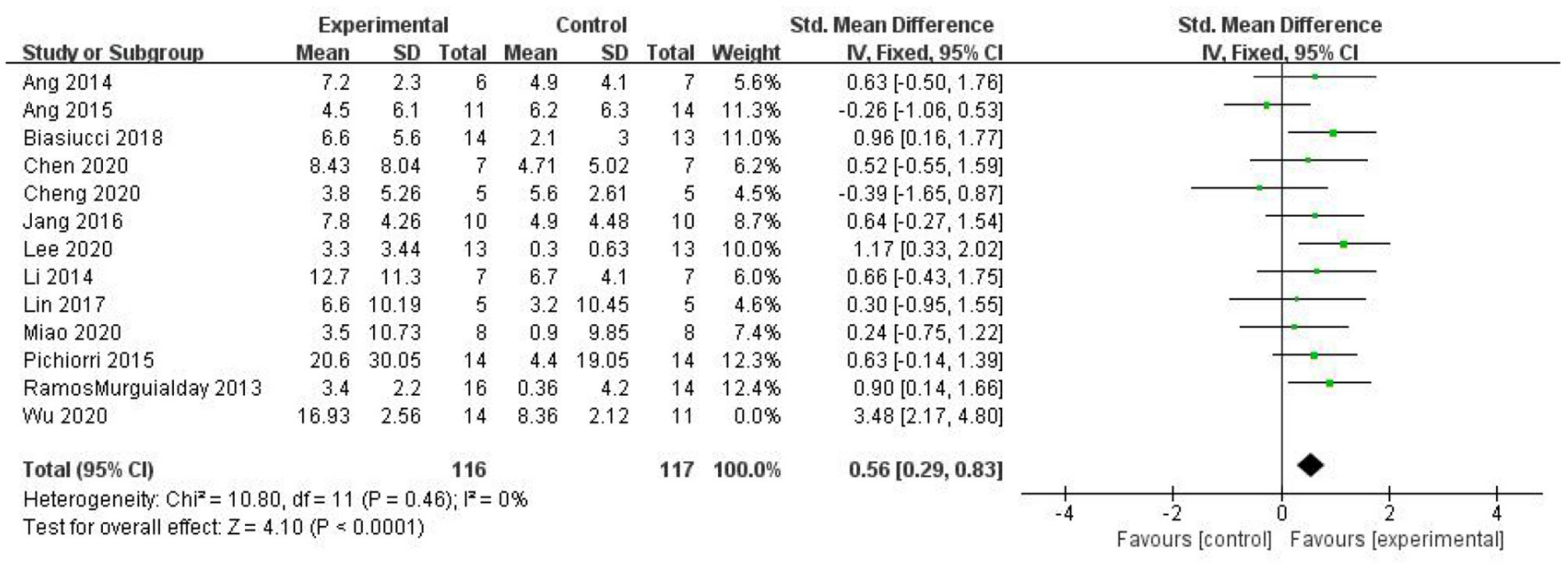
Comparison of the effects of BCI interventions and control interventions on upper limb dysfunction before sensitivity analysis and after sensitivity analysis.

**Figure 7:**
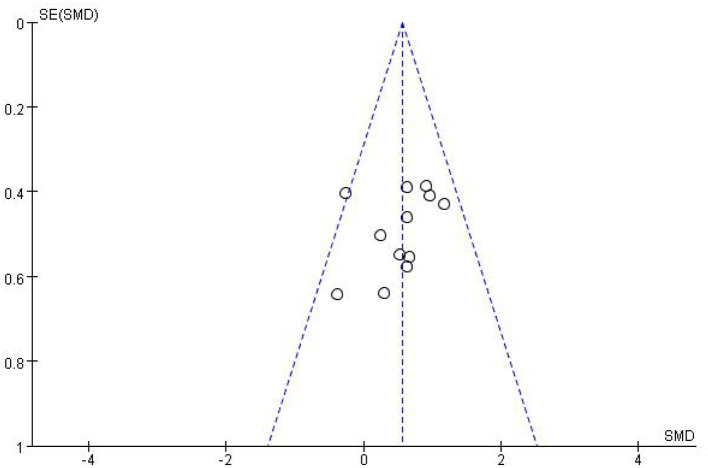
Funnel plot of comparison of the effects of BCI interventions and control interventions on upper limb dysfunction after sensitivity analysis.

Most studies (Ramos-Murguialday et al., 2013; Ang et al., 2014, 2015; Li et al., 2014; Pichiorri et al., 2015; Biasiucci et al., 2018; Lin et al., 2018; Chen et al., 2020; Cheng et al., 2020; Lee et al., 2020; Miao et al., 2020; Wu et al., 2020) used FMA-UE as the outcome measure, and one study (Jang et al., 2016) used MFT as the outcome measure. Because MFT and FMA-UE are continuous variables, we used SMD with 95% CIs to evaluate the pooled results. Results showed that BCI training significantly improved upper limb motor function [SMD = 0.70, 95% CI (0.28, 1.11), *p* < 0.001, random-effects model] (Figure 4). In addition, the studies had significant heterogeneity (*p* < 0.001, *I*^2^ = 59%), and the funnel plot showed an asymmetric state (Figure 5). For the sensitivity analysis, the meta-analysis was conducted again after removing one study at a time to investigate whether the results changed. The sensitivity analysis revealed that the main source of heterogeneity was the study by Wu et al. (2020); after excluding this study, the heterogeneity was reduced significantly (*I*^2^ = 0%). The results of the fixed-effects model showed that BCI training significantly improves upper limb motor function [SMD = 0.56, 95% CI (0.29, 0.83), *p* < 0.001; Figure 6], and the funnel plot became more symmetrical (Figure 7).

#### Subgroup Analysis of the Efficacy of BCI for Different Intervention Durations

**Figure 8:**
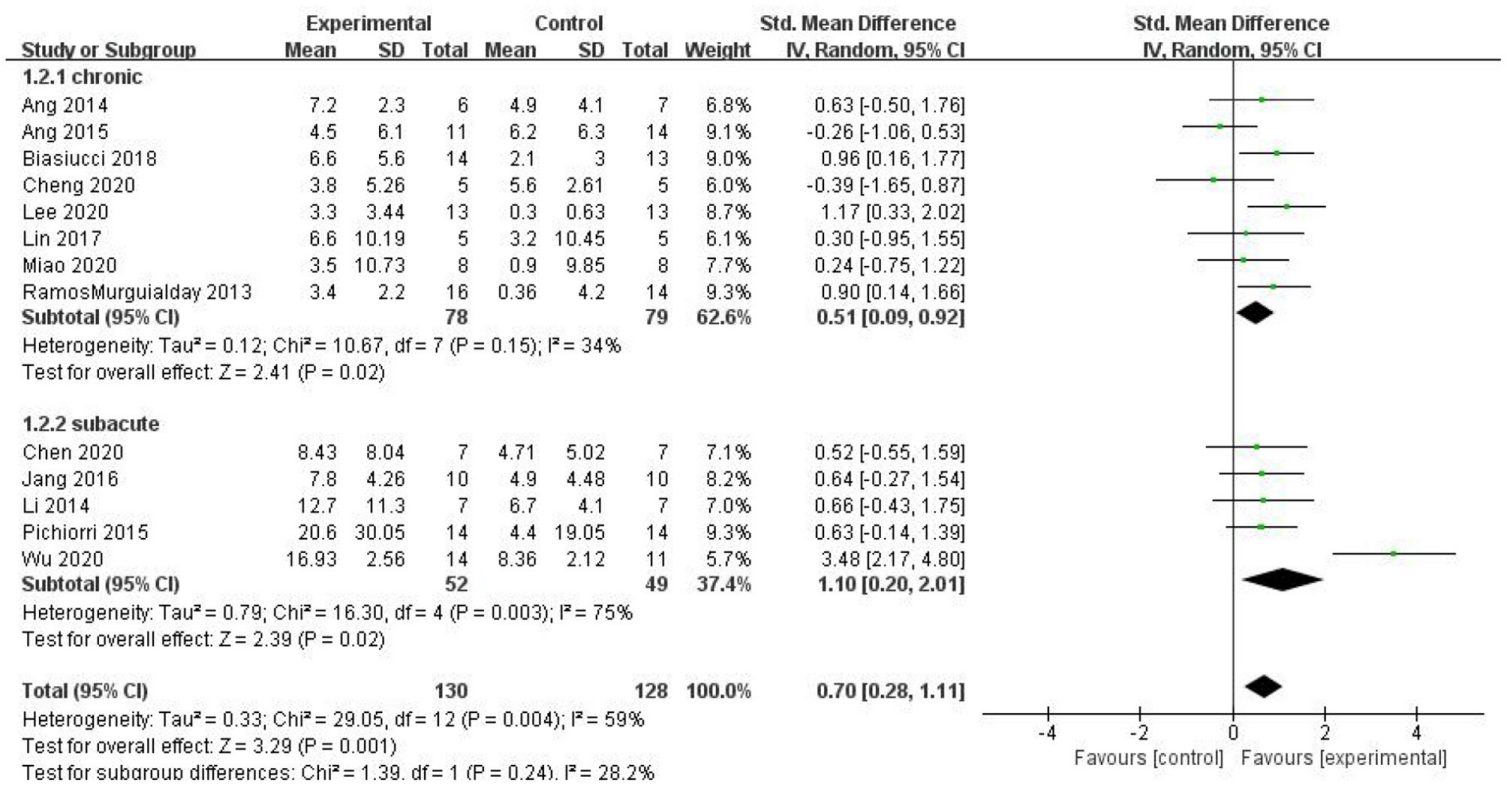
A subgroup analysis of the effects of BCI on upper limb motor function in different intervention periods.

For the subgroup analysis, we used acute or subacute stroke stage as the criterion. For the intervention period following stroke, the duration of onset was limited to 6 months; longer than 6 months was considered the chronic phase, and up to 6 months was considered the subacute phase. The studies by Li et al. (2014), Pichiorri et al. (2015), Jang et al. (2016), Chen et al. (2020), and Wu et al. (2020) were included in the subacute group, and the studies by Ramos-Murguialday et al. (2013), Ang et al. (2014, 2015), Biasiucci et al. (2018), Lin et al. (2018), Cheng et al. (2020), Lee et al. (2020), and Miao et al. (2020) were included in the chronic group. As shown in Figure 8, the results of the subgroup analysis revealed that both the subacute [SMD = 1.10, 95% CI (0.20, 2.01), *p* = 0.02] and chronic groups exhibited a superior effect of BCI on upper limb motor function than that of the control group [SMD = 0.51, 95% CI (0.09, 0.92), *p* = 0.02]. However, the difference between the two subgroups was not significant (*p* = 0.24).

## Discussion

BCI training has recently emerged as a novel method to improve upper limb motor function in stroke patients. Here, we conducted a meta-analysis to investigate the efficacy of BCI training on the limb function of stroke patients.

Our meta-analysis included 258 stroke patients from 13 studies, and results showed that BCI training significantly promotes the recovery of upper limb motor function. Based on our evidence-based medical analysis of relevant studies, we found that BCI is beneficial to the recovery of upper limb function following stroke, which provides support for its clinical application. Our meta-analysis included additional studies to those conducted previously (Cervera et al., 2018; Bai et al., 2020). Because the evaluation time for limb motor function varied from 6 weeks (Ang et al., 2015) to 12–24 weeks (Cheng et al., 2020) across various studies, we investigated immediate evaluation shortly after training rather than follow-up evaluation. In addition to evaluation time, we considered intervention time as another influencing factor for treatment effects. The reviews by Cervera et al. (2018) and Bai et al. (2020) also showed that BCI training improves hand function in stroke patients. However, neither review stated whether intervention time affected efficacy. Here, we performed a subgroup analysis of intervention time, and the results suggested that both the subacute and chronic groups showed significant improvement in upper limb motor function, with the subacute group showing the greatest improvement.

The source of heterogeneity in our study came from the study conducted by Wu et al. (2020), which investigated the clinical efficacy of BCI training and the changes in brain functional networks. In contrast to other studies, patients in this study received more intense training with a total of 20 BCI training sessions (lasting for 1 h per day, 5 days per week, over 4 weeks) and shorter training time intervals between each session, which ensured that patients received sufficient treatment time. This study yielded the best efficacy.

Our findings clearly indicate that BCI training can improve patients' recovery. However, the use of external auxiliary equipment requires patients to focus and cooperate with therapists; the more that patients focus on the training, the greater the effectiveness. The BCI training system acquires, analyzes, and translates brain signals into control commands for output devices when the motor cortex of the brain sends signals indicating the intention to move (Wolpaw et al., 2002; Kim et al., 2016). The neural mechanism of BCI training is mainly attributed to changes in neuroplasticity, which may be reflected by changes in the functional connections and structure of the brain. The studies (Ramos-Murguialday et al., 2013; Li et al., 2014; Pichiorri et al., 2015; Biasiucci et al., 2018; Chen et al., 2020; Wu et al., 2020) included in our meta-analysis used electroencephalography and MRI to analyze the functional connections between hemispheres (including the temporal, parietal, occipital, and subcortical regions), which may partly explain the mechanisms. In addition to changes in brain structure, changes in the integrity of the corticospinal tract may be another contributor to the improvement in motor function. The study by Halder et al. (2013) used magnetic resonance diffusion tensor imaging to visualize structural changes and discovered that changes in the integrity (fractional anisotropy value) of the corticospinal tract of the regions of interest (e.g., the right cingulate, left fronto-occipital tract, corpus callosum, left cerebral infarction, and right posterior coronal radiation) were positively correlated with changes in motor function, which suggested that BCI training improves the integrity of the corticospinal tract to regulate neuroplasticity, and thus facilitates the improvement of motor function.

We chose FMA-UE as the evaluation indicator for motor function. FMA is a quantitative score of patient motor function based on Brunnstrom staging. In addition to FMA-UE, Modified Barthel Index (MBI) and the Modified Ashworth Scale (MAS) were also used as evaluation tools for post-stroke patients. A study conducted by Lee et al. found that MBI changed (7.07 ± 6.31) after BCI training (Lee et al., 2020), whereas another study showed no significant differences between the BCI and control groups (Jang et al., 2016) based on the MAS metric.

A limitation of our analysis is that the number of included patients was small, which may affect the quality of the review results. Because there are considerable variations in the model and implementation methods (especially treatment time) of BCI in clinical use, the efficacy of BCI training varies between individuals. Previous clinical trials seldom mention the follow-up efficacy of BCI training; thus, future studies may investigate the optimal training time to achieve favorable immediate and long-term improvements in hand function in post-stroke patients.

## Conclusions

Our analysis revealed that BCI training significantly improves upper limb motor function in both subacute and chronic stroke patients. Existing studies revealed that the mechanisms of BCI are primarily related to improvements in the functional connectivity and structural integrity of the brain. Further studies are needed to explore optimal training and evaluation times for BCI training.

## Data Availability Statement

The original contributions presented in the study are included in the article, further inquiries can be directed to the corresponding author/s.

## Author Contributions

WY, YH, and XZ contributed to the typographical logic of the article. CX, QZ, and ZL provided help for the data collection of the article. All authors contributed to the article and approved the submitted version.

## Funding

This work was supported by the Longhua District High-level Medical Team Project, the 2021 Basic Research Project of Shenzhen Science, Technology and Innovation Commission (ID: JCYJ20210324123414039), and the Shenzhen Longhua District Rehabilitation Medical Equipment Development and Transformation Joint Key Laboratory in 2021.

## Conflict of Interest

The authors declare that the research was conducted in the absence of any commercial or financial relationships that could be construed as a potential conflict of interest.

## Publisher's Note

All claims expressed in this article are solely those of the authors and do not necessarily represent those of their affiliated organizations, or those of the publisher, the editors and the reviewers. Any product that may be evaluated in this article, or claim that may be made by its manufacturer, is not guaranteed or endorsed by the publisher.
